# Recent advances in pathogenetic concepts and disease modeling of IgA nephropathy

**DOI:** 10.1093/ckj/sfaf152

**Published:** 2025-05-16

**Authors:** Leonie Dreher, Lars Nilges, Thorsten Wiech, Markus M Rinschen, Nicola M Tomas

**Affiliations:** III. Department of Medicine, University Medical Center Hamburg-Eppendorf, Hamburg, Germany; Hamburg Center for Kidney Health (HCKH), University Medical Center Hamburg-Eppendorf, Hamburg, Germany; III. Department of Medicine, University Medical Center Hamburg-Eppendorf, Hamburg, Germany; Hamburg Center for Kidney Health (HCKH), University Medical Center Hamburg-Eppendorf, Hamburg, Germany; Hamburg Center for Kidney Health (HCKH), University Medical Center Hamburg-Eppendorf, Hamburg, Germany; Institute of Pathology, Nephropathology Section, University Medical Center Hamburg-Eppendorf, Hamburg, Germany; III. Department of Medicine, University Medical Center Hamburg-Eppendorf, Hamburg, Germany; Hamburg Center for Kidney Health (HCKH), University Medical Center Hamburg-Eppendorf, Hamburg, Germany; Department of Biomedicine, Aarhus University, Aarhus, Denmark; III. Department of Medicine, University Medical Center Hamburg-Eppendorf, Hamburg, Germany; Hamburg Center for Kidney Health (HCKH), University Medical Center Hamburg-Eppendorf, Hamburg, Germany; Hamburg Center for Translational Immunology, University Medical Center Hamburg-Eppendorf, Hamburg, Germany

**Keywords:** complement, glomerulonephritis, IgA nephropathy, immunosuppression, proteinuria

## Abstract

IgA nephropathy (IgAN) is the most common form of glomerulonephritis and affected patients are at high risk of developing kidney failure over time. Recent molecular studies have led to substantial new insights into the pathogenesis of IgAN. This involves the identification of genetic risk factors in genome-wide association studies, the use of multi-omics approaches to integrate big data, the recognition of the importance of the gut–kidney axis, the role of plasma cells in the production of IgA and IgG, the potential specificity of circulating IgA for mesangial antigens, and the activation of the complement system with subsequent damage to glomerular cells. These fundamental insights were governed by the use of various animal models involving mesangial deposition of IgA, inflammation and glomerular injury. This review summarizes recently identified pathophysiological mechanisms as well as animal models of IgAN and provides an updated view on the molecular events that underlie IgAN.

## INTRODUCTION

IgA nephropathy (IgAN) is the most prevalent primary glomerulonephritis (GN) globally with a high lifetime risk of kidney failure [1]. Recently, multiple studies have led to important discoveries, substantially improving our understanding of key steps involved in the immunopathogenesis of IgAN. Furthermore, large clinical trials have reconfigured the landscape of available therapies for patients with IgAN. In this article, we review emerging developments in the field of clinical and translational IgAN research and describe how these novel findings may influence future strategies to improve the outcome of patients with IgAN.

**Figure 1:**
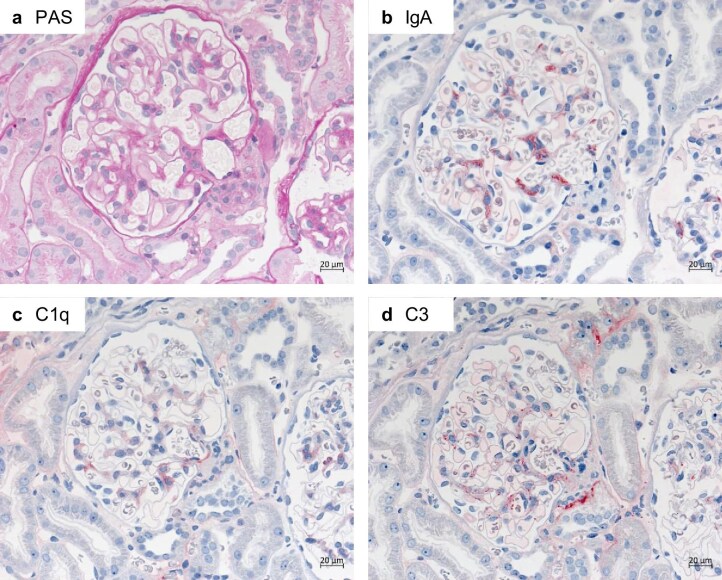
Histopathological pattern of IgA nephropathy. (**a**) Periodic acid Schiff (PAS)-staining shows mesangial expansion and endocapillary hypercellularity. (**b**) Immunohistochemistry for IgA shows a strong mesangial positivity for IgA. (**c, d**) Complement components C1q (c) and C3 (d) show intraglomerular positivity.

IgAN is histologically characterized by mesangial hypercellularity, mesangial matrix expansion, and a variable degree of endocapillary hypercellularity, segmental sclerosis, and tubulointerstitial fibrosis (Fig. 1a) [2, 3]. In rare cases, >50% glomerular crescents may be observed, indicating rapidly progressive GN. Immunohistochemistry typically reveals mesangial IgA deposits (Fig. 1b) as well as mesangial positivity of complement components such as C3 and, often to a lesser degree, C1q (Fig. 1c and d). The prevalence of IgAN exhibits a geographic gradient, with East Asia reporting 40%–50% of all GN cases [4]. Moreover, in Western regions, male patients are more frequently affected (2.3:1 male-to-female ratio) compared to a nearly equal distribution in East Asia [5].

Clinically, IgAN is marked by microhematuria, occasional macrohematuria, proteinuria, gradual loss of kidney function, and, in more severe cases, acute kidney injury or rapid progression to kidney failure [6]. The disease has considerable socioeconomic implications, with up to 64% of patients requiring dialysis/transplantation or dying within 20 years if risk factors such as hypertension, severe proteinuria, and extensive pathological lesions are present [1]. As such, understanding the molecular pathogenesis of IgAN is crucial to developing targeted therapies that can prevent progression to kidney failure.

The widely accepted “4-hit hypothesis” provides a framework for understanding the development of IgAN [2]. This model proposes that the disease begins with the production of galactose-deficient IgA1 (Gd-IgA1), which is thought to be the first hit [2, 7]. These aberrant molecules trigger the formation of anti-glycan antibodies (second hit) [8]. The subsequent immune complex formation (third hit) [9] leads to mesangial deposition and glomerular inflammation (fourth hit), culminating in progressive glomerular scarring [2, 10]. While this model is established, the precise molecular mechanisms underlying each of the four steps remain incompletely understood. Recent advancements in genetics, immunology, and microbiology have provided valuable insights into these processes and are summarized in this review.

## GENETIC BASIS OF IgAN

The genetic architecture underlying IgAN is complex, with familial clustering observed in some cases, suggesting a hereditary component. Elevated levels of Gd-IgA1 are frequently found in the relatives of IgAN patients, although not all develop the disease, indicating that additional genetic factors are involved in the disease's pathogenesis [11]. Genome-wide association studies (GWAS) have proved instrumental in identifying risk loci for IgAN. A major study by Kiryluk *et al.* in 2014 identified 15 risk loci, many of which are linked to the immune system and mucosal barrier function [12]. A follow-up study in 2023 expanded this list to 30 risk loci, of which 16 were newly discovered [13]. Most of these loci are associated with the innate and adaptive immune systems, the complement system, and gastrointestinal mucosal immunity. The findings of these two studies highlight the presence of a polygenetic architecture of IgAN.

**Figure 2:**
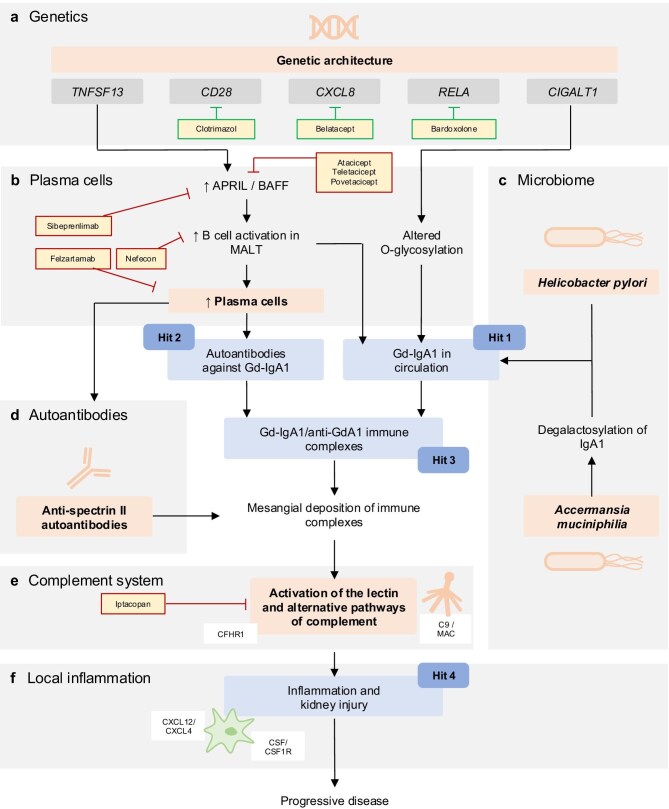
Updated view on the pathogenesis of IgAN. Scheme on the involvement of genetic factors (**a**), plasma cells (**b**), the microbiome (**c**), autoantibodies targeting the mesangium (**d**), the complement system (**e**), and local inflammation (**f**) in the pathogenesis of IgAN. Blue boxes indicate the steps of the classical “4-hit model,” red boxes indicate new pathogenic concepts, yellow/red boxes indicate pathogenesis-based treatments that were recently approved or are currently under investigation, and yellow/green boxes indicate drug candidates prioritized with GWAS support. MALT, mucosa-associated lymphoid tissue; CFHR1, complement factor H-related protein 1; MAC, membrane attack complex; CXCL12, CXC-motif-chemokine 12; CXCL4, CXC-motif-chemokine 4, CSF, colony stimulating factor; CSF1R, colony stimulating factor 1 receptor.

Significant risk loci were further tested in mouse models, where modification of associated genes such as *TNFSF13, TNFSF13B, ITGAM, RELA, REL, CD28*, and *LYN* in mice led to an increase in IgA levels [13]. To evaluate possible drug repurposing, risk loci for genes with already available drugs targeting the encoded protein were filtered. Possible drug targets included blocking of the alternative complement pathway, APRIL (a proliferation-inducing ligand) inhibition, inhibition of CD28, IL8, IL8 receptor, or NFκB (Fig. 2a) [13].

Notably, another GWAS has highlighted genetic variations in *C1GALT1*, a gene encoding a galactosyltransferase responsible for the *O*-galactosylation of IgA [14]. This alteration is thought to contribute to the formation of Gd-IgA1, the first hit in the disease cascade (Fig. 2a). In addition, APRIL, encoded by *TNFSF13*, is involved in the differentiation of B cells into IgA-producing plasma cells, which is essential for the overproduction of Gd-IgA1 (Fig. 2a). These findings suggest that the genetic risk for IgAN is polygenic, with multiple loci contributing to disease onset and progression. Importantly, a high polygenic risk score correlates with earlier disease onset and a higher risk of kidney failure, offering a potential tool for predicting disease course and outcome. In the future, the use of such risk scores could potentially pave the way to a more personalized treatment for IgAN patients. Well-validated polygenetic risk scores may be a helpful tool for predicting disease progression and patient-specific outcomes, possibly allowing for individualized treatment adjustments. Furthermore, the screening for risk loci offers the opportunity to develop a patient-tailored therapeutic approach that specifically targets altered pathways or proteins. Drug repurposing strategies may further enlarge the toolbox of therapies for patients with IgAN.

## PLASMA CELLS AND IgA DYSREGULATION

Plasma cells are central to the pathogenesis of IgAN, primarily through their production of abnormal IgA. As mentioned previously, polymorphisms in *C1GALT1* have been linked to increased levels of Gd-IgA1 (Fig. 2a and b) [14]. A key feature of IgAN is the defective *O-*galactosylation of IgA1, which impairs its clearance from the circulation, leading to accumulation and subsequent mesangial deposition [15]. The mucosal immune system, particularly in the gastrointestinal and respiratory tracts, plays a critical role in this process. Plasma cells in mucosa-associated lymphoid tissue are the primary source of IgA production, which forms dimers or polymers that then enter the bloodstream [16, 17].

Recent findings further emphasize that plasma cells, particularly in the bone marrow and mucosal tissues, play a direct role in producing the pathogenic IgA1 (Fig. 2b). Specifically, plasma cells from IgAN patients demonstrate an increased propensity to switch from producing monomeric IgA (mIgA) to the more pathogenic Gd-IgA1 [16, 18]. Translational genetic studies in familial and sporadic IgAN identified rare GALNT14 loss-of-function mutations in patients, with functional validation in a murine knockout model demonstrating impaired B-cell *O*-glycosylation and homing [19]. These findings implicate glycosylation defects in IgAN pathogenesis independent of the classic hinge-region galactose deficiency of IgA1. This not only supports B cells as a therapeutic target, it also raises the possibility that undergalactosylated IgA may serve more as a bystander than as a primary disease driver.

Further elucidating the pathogenesis of IgAN, recent studies have highlighted the role of APRIL, a cytokine involved in B-cell maturation (Fig. 2b) [20]. Increased activity of APRIL has been linked to the disease, with elevated levels of APRIL correlating with disease severity [20]. In clinical trials, the use of APRIL inhibitors such as sibeprenlimab is being explored as a potential therapeutic approach to modulate the excessive IgA production in IgAN [21]. The B-cell-activating factor (BAFF) is also essential for B-cell proliferation and antibody production. Studies showed elevated serum levels of BAFF in patients with IgAN [22]. Thus, plasma cells represent a promising therapeutic target, and interventions aimed at modulating plasma cell function or IgA production may offer new treatment avenues for IgAN.

## THE ROLE OF THE MICROBIOME

A role of an altered microbiome in the pathogenesis in IgAN has long been postulated. Recent studies suggest that dysbiosis, or an imbalance in gut microbiota, may contribute to the disease. Bacteria in the intestinal flora can modify immunoglobulins, particularly IgA, through enzymatic processes such as degalactosylation [23]. This modification of IgA1 can lead to an immune response that triggers the formation of anti-Gd-IgA1 antibodies, which may then form immune complexes that deposit in the glomeruli.

In animal models, depletion of the gut microbiome has been shown to prevent the development of IgAN, highlighting the significance of the gut–kidney axis in disease progression [24]. Notably, the presence of *Akkermansia muciniphila*, a bacterium associated with mucosal health, has been linked to IgA dysregulation in IgAN patients [25]. A recent study has demonstrated that this bacterium can degalactosylate IgA, potentially contributing to the formation of Gd-IgA1 (Fig. 2c). The altered microbiome may also enhance the immune system's response to intestinal pathogens, such as *Helicobacter pylori*, which can exacerbate IgAN by promoting abnormal IgA production and immune complex formation (Fig. 2c) [25].

These findings suggest that modulating the microbiome may offer a novel therapeutic strategy for IgAN. Manipulating gut flora or using probiotics to restore a healthy microbiome could potentially attenuate the immune dysregulation that underpins IgAN.

## AUTOANTIBODIES AND IMMUNE COMPLEX FORMATION

The formation of immune complexes in IgAN is a hallmark of the disease, with Gd-IgA1 being the primary component. These complexes deposit in the mesangium, triggering an inflammatory cascade that results in glomerular damage. Recent studies suggest that the formation of these immune complexes may not occur in the circulation, but rather *in situ* in the mesangium [26].

Based on this assumption, an elegant experimental study recently demonstrated the presence of IgA autoantibodies in the sera of mice subjected to an IgAN mouse model [27]. The target antigen of these IgA autoantibodies was βII-spectrin (Fig. 2d). ΒII-spectrin is a ubiquitous protein also expressed in the human mesangium. In this study, the authors found anti-βII spectrin autoantibodies in ∼60% of IgAN patients [27]. By contrast, they were very rarely found in healthy individuals [27]. Using the grouped ddY mouse model of IgAN (Table 1), IgA1-producing plasma blasts were detected in the kidney of the diseased mice. Secretion of anti-βII spectrin autoantibodies by these plasma cells was further confirmed [27]. However, the origin of these plasma cells was not conclusively clarified. Priming of the B cells in the (intestinal) mucosa with subsequent migration to the kidney is a possible mechanism [27]. Understanding the mechanisms behind these autoantibodies and their role in immune complex formation could provide new insights into IgAN pathogenesis and open avenues for targeted therapies. Felzartamab, a monoclonal antibody targeting CD38 is an emerging treatment strategy in IgAN. Targeting and depleting autoantibody producing plasma cells could potentially provide an elegant and pathophysiogically based treatment approach in patients with IgAN. A phase 2 trial (NCT05065970) for the use of felzartamab in IgAN was recently completed.

**Table 1:** Overview of selected IgAN mouse models.

Name	Mechanism of IgAN phenotype induction	Phenotype	Advantages	Limitations	Source
DNP model	Mesangial deposition of *in vitro* or *in vivo* formed DNP-BSA-IgA IC	Proteinuria, hematuria, induction of glomerulonephritis	First IgAN mouse model to study pathophysiological features	Reduced translation due to *in vitro* model, mouse IgA	[46]
Spontaneous ddY model	Spontaneous mesangial IgA deposition	Mesangial proliferation, induction of glomerulonephritis	Spontaneous development of IgA like phenotype	High variability in disease onset and severity, mouse IgA	[47]
Grouped ddY model	Interbreeding of ddY mice resulting in robust disease onset	s.o.	Robust and reliable onset of disease with 8 weeks of age, altered IgA glycosylation comparable to human phenotype	Mouse IgA	[48]
CD89 transgenic model	Transgenic mice expressing myeloid IgA Fc receptor (CD89)	Spontaneous development of mesangial IgA deposition, mesangial matrix expansion, hematuria, and proteinuria	Increased translation due to expression of human IgA receptor	High variability in disease onset and severity, unclear role of CD89, mouse IgA	[49]
α1KICD9Tg transgenic model	Transgenic expression mice expressing CD89 and human IgA1	Mesangial deposits of IgA1–sCD89 complexes, kidney inflammation, hematuria, and proteinuria	Robust and severe IgAN-like phenotype, human IgA1 expression	Role of CD89 remains unclear	[50]
BAFF model	Transgenic BAFF overexpression	Increased serum IgA levels, increased IgA levels in the intestinal lamina propria and deposition of IgA immune complexes in the mesangium	Induction of a hyper-IgA syndrome	Sex- and gut microbiota- dependent, mouse IgA	[52]
Galnt14 null model	Defective *O*-glycosylation	Increased IgA levels with altered IgA distribution, glomerular IgA deposition	Defect in O-glycosylation, gut mucosal phenotype	Mouse IgA	[19]
Uteroglobin model	Transgenic uteroglobin deficient mice resulting in IgA-fibronectin IC	Hematuria, mesangial IgA and C3 deposition	Proving of binding ability of IgA to non-antigenic proteins	No proteinuria and normal kidney function, poor reproducibility of the model, mouse IgA	[56]
Bcl-2 model	Transgenic overexpression of Bcl-2	IgA hyperglobulinemia and glomerulonephritis	Altered glycosylation profiles comparable to human IgAN	Mouse IgA	[53]
LIGHT model	LIGHT overexpressing transgenic mice with activation of IgA-producing B cells in the intestine	IgA and C3 mesangial deposition, proteinuria, and hematuria	Highlighting importance of T-cell mediated B-cell activation in IgAN	Mouse IgA	[55]
β4GalT-I KO model	β-1,4 galactosyltransferase deficient mice	High IgA serum levels, mesangial deposition of IgA	absence of galactosylation and sialylation of IgA	Mouse IgA	[54]

## COMPLEMENT SYSTEM ACTIVATION

Complement activation is a critical mediator of glomerular injury in IgAN. The complement system can be activated through three primary pathways: classical, alternative, and lectin. In IgAN, the alternative and lectin pathways appear to be particularly relevant (Fig. 2e) [28]. The alternative pathway is triggered by the spontaneous hydrolysis of C3, leading to the formation of the C3 convertase [29]. The lectin pathway is activated by pattern recognition molecules binding to mannose-binding lectin and associated serine proteases [30].

Immunohistochemical studies have shown that complement components, particularly C3, are deposited in the glomeruli of up to 90% of IgAN patients [30], with the intensity of C3 deposition correlating with disease severity [28]. The lectin pathway, in particular, is implicated in glomerular injury, with components such as MBL and C4d detected in a significant proportion of IgAN cases. The presence of these components is associated with worse histological outcomes and clinical parameters, such as proteinuria and renal function decline [31, 32]. Therefore, the degree of complement deposition in kidney biopsies can be used as a surveillance marker in IgAN patients. Further, some studies suggest a correlation between serum C3 to IgA ratio with disease severity [33, 34].

The alternative pathway is similarly involved, with factor B, factor H, and FHR proteins being present in up to 97% of IgAN cases [30, 35]. Studies have shown that deficiencies or deletions in complement regulatory proteins, such as CFHR1 and CFHR3, offer a protective effect against IgAN [11, 12]. These findings emphasize the importance of complement regulation in the disease and suggest that complement inhibition could be a promising therapeutic approach.

## USE OF OMICS APPROACHES IN IgAN

Recent advances in transcriptomics and proteomics have greatly expanded the understanding of IgAN pathogenesis and thus opened new opportunities for drug discovery and development. Single-cell RNA sequencing has identified inflammatory signaling pathways, including CXCL12/CXCR4 and CSF1/CSF1R in mesangial cells and macrophages (Fig. 2f), as major drivers of disease progression, while endothelial dysfunction, glomerulo-tubular crosstalk, were also implicated [36]. Furthermore, spatial transcriptomic analysis of IgAN patient biopsies distinguished proliferative from non-proliferative IgAN and identified TCF21 as a shared marker of podocyte injury, implicating cell-type-specific pathways in disease progression [37]. Targeting the CXCL12/CXCR4 axis reduced glomerular inflammation, complement deposition, and fibrosis in both BAFF-transgenic mice (Table 1) and anti-Thy1.1 nephritis rats, supporting its therapeutic potential in IgAN [36].

Non-coding RNAs and blood transcriptomics of patient samples further enriched biomarker discovery. Urinary exosomal miR-204, although lacking disease specificity alone, improved prognostic accuracy when combined with clinical risk scores, supporting its potential in patient stratification [38]. Further, elevated levels of exosomal miR-483–5p correlate with proteinuria and kidney failure in IgAN patients and promote a proinflammatory phenotype in collecting duct epithelial cells via SOCS3 suppression [39]. In addition, transcriptomic profiling from peripheral blood mononuclear cells identified KLRC1 and C1QB as novel potential diagnostic biomarkers and proposed candidates for immunomodulatory therapies [40].

The use of proteomics has provided additional important insights. Proteomic profiling of human glomeruli revealed enrichment of immunoglobulins, complement factors, and extracellular matrix proteins in IgAN. Interestingly, the study described a strong accumulation of the terminal membrane attack component C9 and complement-H-factor related protein 1 (CFHR1) as disease-specific markers (Fig. 2e) [41]. A multiancestry proteome-wide Mendelian randomization and colocalization study identified complement regulators (CFHR1, CFH) and the B-cell signaling molecule FCRL2 as putative causal contributors to IgAN, aligning with and supporting current therapeutic strategies targeting the complement cascade and B-cell pathways [42]. In addition, urinary proteomic studies identified dysregulated complement components (e.g. CFB, CFH, CFHR2) strongly associated with disease severity, suggesting urine-based complement profiling could guide diagnosis and therapy [43].

Collectively, these studies underscore the power of integrated multi-omics approaches in unraveling the pathogenesis of IgAN and highlight their potential to guide the development of novel biomarkers for patient risk stratification and targeted therapeutic strategies.

## ANIMAL MODELS OF IgA NEPHROPATHY: APPLICATIONS AND LIMITATIONS

Animal models are indispensable for advancing our understanding of the pathogenesis of autoimmune kidney diseases, including IgAN. These models help elucidate key mechanisms involved in disease initiation and progression, enabling the development of novel therapeutic strategies. However, despite their utility, several pitfalls remain, particularly when modeling a complex and chronic condition like IgAN. Differences in the characteristics and functions of IgA between species, particularly between humans and mice, represent one such challenge.

Humans express two distinct isotypes of IgA, namely IgA1 and IgA2, which differ in both structure and function. In contrast, mice produce a single form of IgA that is more similar to human IgA1 but still differs in several respects, including the glycosylation patterns [44, 45]. These interspecies differences can limit the direct applicability of mouse models for studying IgAN, particularly with respect to the glycosylation of IgA, which is central to disease pathogenesis. In addition, while IgAN in humans is typically a chronic and slowly progressive disorder, such progression is difficult to replicate in animal models, as the duration of the disease in mice is often too short to accurately model long-term outcomes.

**Figure 3:**
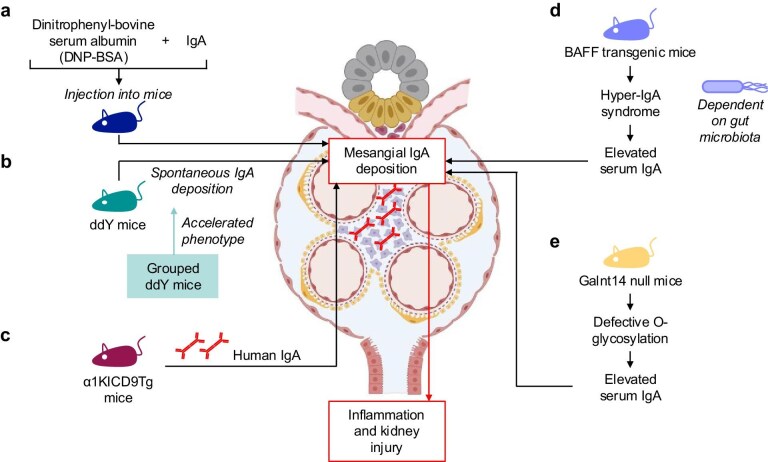
Animal models of IgAN. Several models of IgA have been developed over the last decades, involving, among others, the DNP-BSA model (**a**), gddY and grouped gddY mice (**b**), α1KICD9Tg mice (**c**), BAFF-transgenic mice (**d**), and Galnt14 null mice (**e**).

The earliest animal models of IgAN involved the use of immune complexes. These included *in vitro* or *in vivo* formed immune complexes, such as dinitrophenyl-bovine serum albumin (DNP-BSA) and IgA, which were administered to mice (Table 1 and Fig. 3a) [46]. The resulting immune complexes led to mesangial deposition and subsequent renal damage, recapitulating some aspects of the human disease [46]. While these models provided valuable insights into the pathophysiology of IgAN, they failed to mimic the spontaneous, chronic nature of the disease, making them less effective for studying long-term progression.

A significant breakthrough came in 1985 with the identification of the ddY mouse model, which spontaneously develops IgA deposits in the mesangium, mimicking some of the key features of IgAN (Table 1 and Fig. 3b) [47]. The ddY mouse is characterized by the spontaneous deposition of IgA in the glomeruli, a hallmark of IgAN. Seven years later, the grouped ddY model was introduced to accelerate the development of the disease phenotype (Table 1) [48]. In this model, mice from the ddY strain that developed IgAN early in life were selectively bred, resulting in offspring with a more robust onset of disease at ∼8 weeks of age. These mice exhibit typical clinical and histological features of IgAN, including glomerular IgA deposition, mesangial hypercellularity, and proteinuria [48].

Notably, like human IgAN, the grouped ddY model also demonstrates alterations in IgA glycosylation, particularly the reduced galactosylation of IgA1, which is commonly observed in IgAN patients [48]. This resemblance to the human disease makes the ddY model particularly valuable for studying IgAN pathogenesis, particularly in the context of glycosylation abnormalities and their contribution to disease progression. However, the previously mentioned differences between mouse and human IgA remain a limitation in regard to translatability of the results.

To overcome the interspecies differences in IgA, humanized mouse models have been developed. These models involve the expression of human IgA or human IgA receptors in mice, allowing for a closer approximation of the human disease. One such model, developed in 1990, involved the generation of transgenic mice expressing CD89, the myeloid Fc receptor for IgA [49]. These mice were designed to examine the role of CD89 in IgAN, as it plays a critical role in the clearance of immune complexes from the circulation. However, various transgenic CD89 models have shown varying phenotypes of IgAN, likely due to differences in molecular regulation and model design [45].

A further refinement of this model involved the development of the α1KICD9Tg mouse by Monteiro and colleagues. These mice express both CD89 and human IgA1, allowing for the study of IgAN pathogenesis in a system that more closely resembles human disease (Table 1 and Fig. 3c) [50]. This model has proven useful for investigating several aspects of IgAN, including the impact of the gut microbiome on the generation of galactose-deficient IgA1 (Gd-IgA1) [25]. Additionally, the α1KICD9Tg mouse has been employed to explore the therapeutic potential of IgA1 proteases, which can reduce mesangial IgA deposition and improve disease outcomes in this model [51]. This transgenic model provides a more accurate platform for studying human IgA1 and its role in IgAN, making it an essential tool for evaluating therapeutic interventions targeting human IgA1.

Another approach to modeling IgAN in mice involves the overexpression of BAFF, a cytokine involved in B-cell survival and differentiation. Transgenic mice overexpressing BAFF develop a hyper-IgA syndrome characterized by elevated serum IgA levels and IgA deposition in the glomeruli (Table 1 and Fig. 3d) [52]. These mice exhibit clinical and histological features similar to those seen in IgAN patients, including proteinuria and glomerular damage. The BAFF model has been valuable for studying the role of B-cell dysregulation and IgA hyperproduction in IgAN, as well as exploring potential therapeutic strategies targeting BAFF or its receptor. Of notice, the BAFF model is dependent on the gut microbiota that can potentially limit comparability of results from different study sites.

Most recently, a new animal model for studying IgAN was introduced by Prakash and colleagues. The Galnt14 null mice showed elevated serum IgA level and spontaneously developed glomerular IgA depositions with aging or after induction of sterile colitis (Table 1 and Fig. 3e) [19]. The authors propose, that this mouse model provides a tool to study *O*-glycosylation defects independently of the IgA1 hinge region, since it is absent in mice IgA.

Several other transgenic models have been developed to explore various aspects of IgAN pathophysiology. These include the uteroglobin model, the Bcl2 model, the LIGHT model, and the β4GalT-I knockout (KO) model [52–55]. Each of these models has its own unique advantages and limitations, allowing researchers to investigate different facets of IgAN, such as cytokine signaling, apoptosis, and glycosylation abnormalities.

- The uteroglobin model involves the overexpression of uteroglobin, a protein that modulates immune responses and inflammation. This model has been used to study the role of inflammatory mediators in IgAN pathogenesis [56].
- The Bcl2 model focuses on the role of apoptosis in IgAN. Bcl2 overexpression in B cells leads to enhanced cell survival and excessive IgA production, promoting the development of IgAN-like glomerular injury [53].
- The LIGHT model involves the expression of the LIGHT protein, a member of the tumor necrosis factor (TNF) superfamily. LIGHT has been implicated in the activation of T and B cells, contributing to glomerular inflammation in IgAN [55].
- The β4GalT-I KO model targets β4-galactosyltransferase I, an enzyme involved in the *O*-galactosylation of IgA. Mice lacking this enzyme exhibit defects in IgA glycosylation and develop features of IgAN, making this model useful for studying the impact of glycosylation defects on disease progression [54].

Each of these models has provided valuable insights into the molecular mechanisms underlying IgAN, although they vary in their clinical presentation, disease onset, and severity.

## PATHOPHYSIOLOGY-BASED THERAPEUTIC APPROACHES IN IgAN

The management of IgAN traditionally involves supportive care, which includes lifestyle modifications, blood pressure control, and the use of renin–angiotensin–aldosterone inhibitors and sodium-glucose co-transporter-2 (SGLT2) inhibitors, as recommended by the KDIGO guidelines (2021). While immunosuppressive therapy, particularly corticosteroids, has been shown to benefit high-risk patients, their use is often limited by significant side effects. However, the landscape of IgAN treatment is rapidly evolving, with a growing body of pathophysiological knowledge driving the development of targeted therapeutic interventions (reviewed in [57]). Recent clinical trials have brought new treatments to the forefront, expanding options for managing this challenging disease.

### Targeting B cells and plasma cells

B-cell-targeted therapies have been explored in IgAN, given the crucial role of B cells in the production of pathogenic Gd-IgA1 and potentially autoantibodies targeting mesangial antigens. The B-cell-depleting antibody rituximab was tested in a small randomized controlled trial involving 34 patients with IgAN. However, no significant improvement in renal outcomes were observed [58]. This lack of efficacy may be explained by the presence of plasma cells, which escape rituximab depletion due to their low CD20 expression.

Felzartamab, an anti-CD38 antibody that targets plasma cells (Fig. 2b), is currently being investigated in a phase 2A clinical trial for IgAN, with promising early results [59]. In addition, sibeprenlimab, a monoclonal antibody that binds to APRIL (a cytokine involved in B-cell survival), has shown positive effects on proteinuria in IgAN patients in a phase 2 study [21] (Fig. 2b). These findings suggest that more specific targeting of plasma cells and their survival pathways may offer a promising therapeutic strategy in IgAN.

As elevated level of BAFF and APRIL are found in IgAN patients, dual BAFF/APRIL inhibitors are an interesting therapeutic tool in the treatment of IgAN to directly target the Gd-IgA-producing plasma cells. Inhibition of both factors could advance over single inhibition by preventing compensatory upregulation of the unblocked factor. The dual inhibitor Atacicept was evaluated in a phase 2b study, the ORIGIN trial [60]. Results showed a reduction in proteinuria and a preserved eGFR in the treatment group compared to placebo [60]. Povetacicept, another dual inhibitor of BAFF and APRIL, is currently investigated in IgAN patients in a phase 2 trial (NCT05732402).

### Gut–kidney axis: targeting the mucosal immune system

Recent insights into the role of the gut–kidney axis in the pathogenesis of IgAN have led to the development of novel therapeutic approaches aimed at targeting the mucosal immune system. One such innovation is a new formulation of budesonide, designed for maximum drug release in the distal ileum (Fig. 2b). This formulation specifically targets the mucosal immune cells involved in the production of galactose-deficient IgA1 (Gd-IgA1), a key driver of IgAN pathogenesis. The NefIgArd study, which evaluated this budesonide formulation, demonstrated significant therapeutic effects, including a reduction in proteinuria and a slower decline in GFR in the treatment group [61]. This approach highlights the growing recognition of the gut's critical role in IgAN and the potential benefits of targeting intestinal immune pathways to reduce kidney damage.

### Targeting the complement system

Complement activation plays a central role in the pathogenesis of IgAN, particularly through the alternative complement pathway, which promotes inflammation and kidney injury. Targeting complement activation has emerged as a key therapeutic strategy in IgAN. One such approach involves the inhibition of factor B, a critical component of the alternative pathway (Fig. 2e). The factor B inhibitor iptacopan has shown promising results in clinical trials. In phase 2 studies, iptacopan significantly reduced proteinuria and slowed the progression of kidney disease in IgAN patients [62]. These results were further corroborated by the interim analysis of the ongoing phase 3 APPLAUSE trial, which demonstrated continued efficacy in reducing proteinuria [63]. Notably, the alternative pathway's involvement in IgAN suggests that indirect inhibition through factor B blockade could also minimize the risk of infection-related complications, a concern associated with direct complement inhibition [64]. Based on the promising interim results, the US FDA granted accelerated approval for iptacopan in 2024, marking a significant step forward in the treatment of progressive IgAN.

### Challenges and future directions

While significant progress has been made, several challenges remain in the development and implementation of pathogenesis-based treatments for IgAN. Notably, SGLT2 inhibitors were not a prerequisite in most clinical trials, and renal biopsies are still infrequently performed before initiating treatment with investigational drugs. Although kidney biopsy is a common procedure in clinical practice, its role in large-scale clinical trials remains limited. The routine use of pre-treatment biopsies could provide valuable insights into the histological features of IgAN, such as the degree of mesangial expansion, crescent formation, and the extent of glomerular inflammation and fibrosis. This approach may help identify histological markers that predict treatment response and guide therapeutic decision-making, particularly as more drugs become available for IgAN. The ability to integrate histological data into treatment algorithms will be crucial in ensuring that patients receive the most effective therapy for their individual disease phenotype. Especially as the number of approved treatments increases and new strategies and targets for the treatment of IgAN continue to emerge, it is crucial to develop individualized, biomarker driven treatment strategies for the individual IgAN patient.

## CONCLUSION

The pathogenesis of IgAN involves complex interactions between genetic, immunologic, and environmental factors. While the 4-hit hypothesis provides a conceptual framework, recent advancements have underscored the multifactorial nature of the disease. Genetic predisposition, abnormal IgA production by plasma cells, microbiome dysbiosis, autoantibody formation, and complement activation all contribute to disease progression. Targeted therapies that modulate these processes hold promise for improving the outcomes of IgAN patients, but further research is required to fully understand the molecular mechanisms and to develop additional effective treatments. Understanding the intricate mechanisms underlying IgAN will be crucial for developing precision medicine strategies that can halt or even reverse disease progression, reducing the burden of this oftentimes debilitating condition.

## Data Availability

No new data were generated or analyzed in support of this research.
